# The effects of a bodybuilding thermogenic supplement in conjunction with a periodized resistance training program in resistance trained males

**DOI:** 10.1186/1550-2783-12-S1-P23

**Published:** 2015-09-21

**Authors:** Bill Campbell, Danielle Aguilar, Ryan Colquhoun, Chris Gai, Nic Martinez, Danny Bove, Martin Szauer, Brett Harris, Michael Dumala, Stephen Beaugrand, Kathryn Raines

**Affiliations:** 1 University of South Florida, Performance & Physique Enhancement Laboratory, Tampa, FL, USA

## Background

Males looking to improve their body composition may ingest thermogenic supplements for the purpose of losing body fat. The purpose of this study was to examine the effects of a commercially available dietary supplement (Arnold Iron Cuts™, which contains ingredients that promote thermogenesis) on body composition and maximal strength in a randomized, double-blind, placebo-controlled parallel groups design.

## Methods

34 resistance trained male subjects (21 ± 3.8 years; 177 ± 6.2cm; 79 ± 11.2kg) participated in this investigation. At baseline and following 8-weeks of a periodized resistance-training program, participants were assessed for body composition (fat mass, body fat %, and lean body mass) and maximal strength (back squat and bench press 1RM). After baseline testing, participants were matched according to total fat mass and randomized to the thermogenic supplement group (n = 18) or the placebo group (n = 16). Body composition was assessed via ultrasound and measurements were made using an A mode, 2.5-MHz transmitter (BodyMetrix, Intelametrix). The periodized resistance-training program consisted of whole body workouts conducted three times per week (for 7 weeks) and one week in which two whole-body workouts were conducted. Data were analyzed via a 2-factor [2x2] between-subjects repeated measures analysis of variance (ANOVA) using SPSS version 22.0. The criterion for significance was set at p ≤ 0.05.

## Results

No differences existed between the two groups for any strength or body composition measures at baseline. The repeated measures ANOVA revealed a significant group × time interaction for body fat % (p = 0.044) favoring the thermogenic supplement treatment. Specifically, body fat percentage decreased from 11.9% to 11.0% and 11.8% to 11.7% in the thermogenic and placebo treatments, respectively. There were also changes in total fat mass that resulted in a significant group × time interaction (p = 0.032). Fat mass decreased from 9.6 to 8.9 kg in the thermogenic supplement group and remained stable in the placebo group (slightly increasing from 9.6 to 9.8kg). Lean body mass increased in both groups, increasing from 68.8 to 69.9kg in the thermogenic supplement group and from 69.7 to 70.8kg in the placebo group. These changes resulted in a significant main effect for time (p < 0.001), but no group × time interaction was observed (p = 0.947). Relative to maximal strength, the repeated measures ANOVA revealed a significant main effect for time relative to bench press 1RM (p < 0.001), but no group × time interaction was observed (p = 0.303). Similarly, a main effect for time was observed for squat 1RM (p < 0.001), but no group × time interaction was observed (p = 0.299).

## Conclusions

Resistance trained males engaging in an 8-week periodized resistance training program and consuming a commercially available thermogenic dietary supplement (Arnold Iron Cuts™) can lose a significant amount of fat mass as compared to a placebo treatment. The loss of fat mass can occur while the participants are maintaining normal dietary intakes (i.e., without embarking on a hypoenergetic diet). The thermogenic dietary supplement did not augment gains in lean body mass and offered no advantages related to maximal upper and lower-body strength.

